# Supplementary material for: Quantification of Polyphenols and Metals in Chinese Tea Infusions by Mass Spectrometry
Source: Foods. 2020 Jun 25;9(6):835. doi: 10.3390/foods9060835 (PMC7353651; doi:10.3390/foods9060835)
Supplement: Supplementary file 1 [file foods-09-00835-s001.zip › foods-817165 supplementary.pptx]

## Slide 1
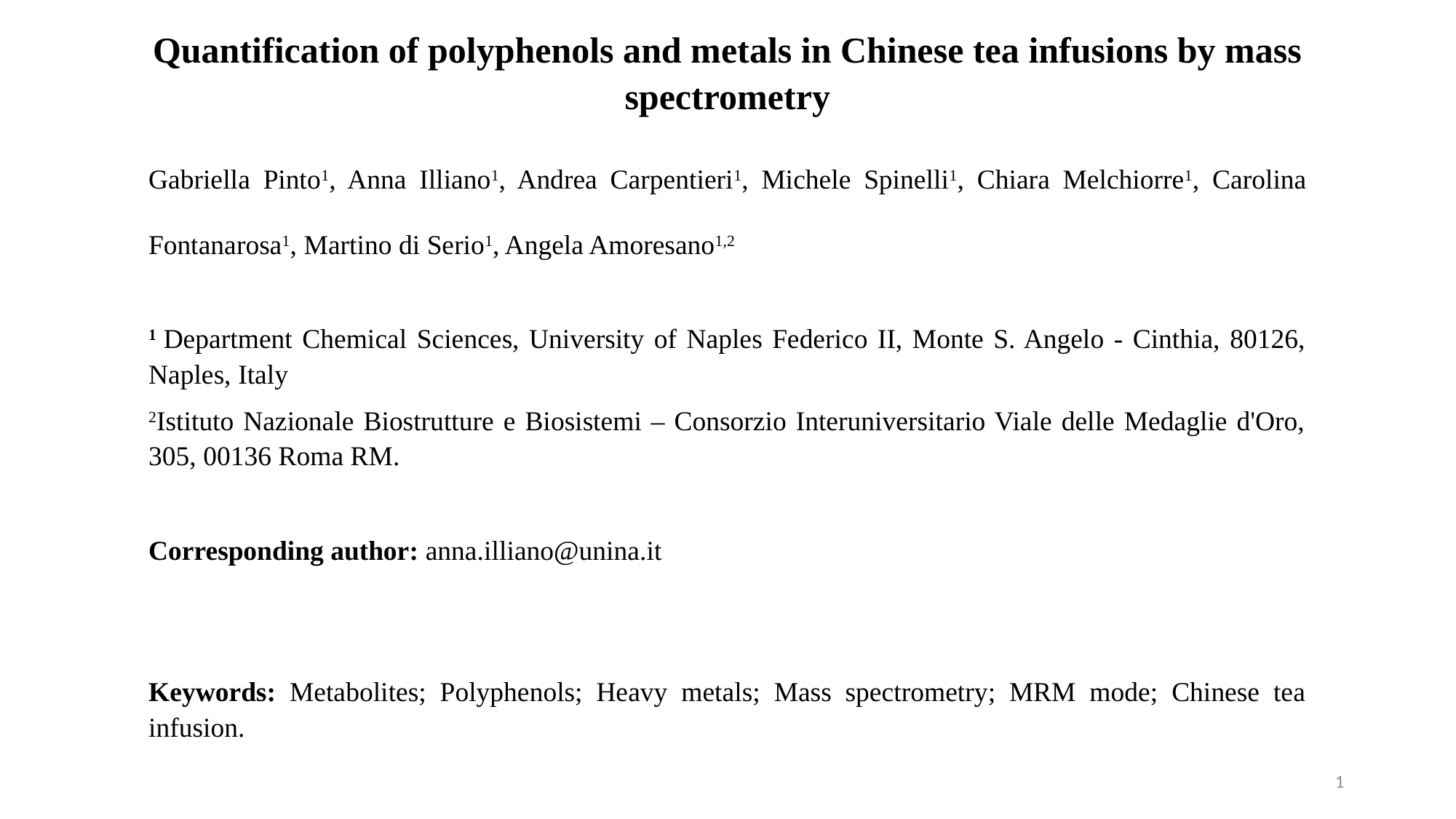

Quantification of polyphenols and metals in Chinese tea infusions by mass spectrometry
Gabriella Pinto1, Anna Illiano1, Andrea Carpentieri1, Michele Spinelli1, Chiara Melchiorre1, Carolina Fontanarosa1, Martino di Serio1, Angela Amoresano1,2
1 Department Chemical Sciences, University of Naples Federico II, Monte S. Angelo - Cinthia, 80126, Naples, Italy
2Istituto Nazionale Biostrutture e Biosistemi – Consorzio Interuniversitario Viale delle Medaglie d'Oro, 305, 00136 Roma RM.
Corresponding author: anna.illiano@unina.it
Keywords: Metabolites; Polyphenols; Heavy metals; Mass spectrometry; MRM mode; Chinese tea infusion.
1

## Slide 2
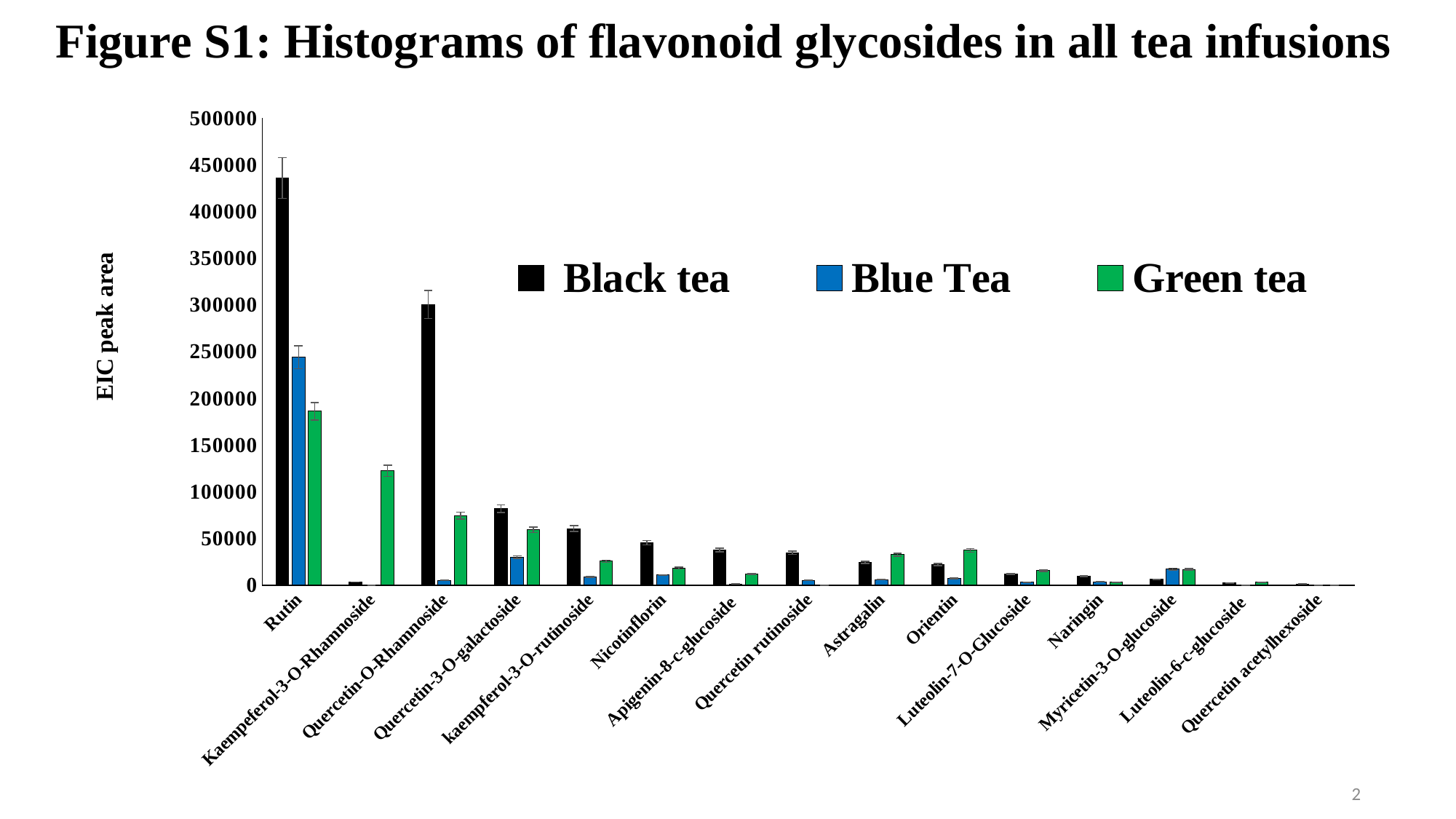

Figure S1: Histograms of flavonoid glycosides in all tea infusions
### Chart
| Category | Black tea | Blue Tea | Green tea |
|---|---|---|---|
| Rutin | 435702.5409708 | 243922.6203135 | 186157.2759135 |
| Kaempeferol-3-O-Rhamnoside | 3267.7750962 | 0.0 | 122403.7182885 |
| Quercetin-O-Rhamnoside | 300206.1219462 | 4951.3780635 | 74315.2434135 |
| Quercetin-3-O-galactoside | 81855.5740335 | 29956.1253135 | 59064.0571634999 |
| kaempferol-3-O-rutinoside | 60511.4605962 | 8919.3858135 | 25502.4496635 |
| Nicotinflorin | 45413.2360962 | 10700.9403135 | 18304.2496635 |
| Apigenin-8-c-glucoside | 37594.1913462 | 1080.6 | 11596.4270385 |
| Quercetin rutinoside | 34644.7288962 | 4960.3758135 | 0.0 |
| Astragalin | 24390.8929962 | 5905.1395635 | 32520.6946635 |
| Orientin | 21978.287641875 | 7137.8313135 | 37199.5246635 |
| Luteolin-7-O-Glucoside | 11819.2366962 | 2935.8820635 | 15510.4482885 |
| Naringin | 9518.4870885 | 3502.7403135 | 3201.5262885 |
| Myricetin-3-O-glucoside | 6045.521829375 | 17089.3428135 | 16864.6096635 |
| Luteolin-6-c-glucoside | 2344.490601 | 0.0 | 3044.0656635 |
| Quercetin acetylhexoside | 1163.5359135 | 0.0 | 0.0 |2

## Slide 3
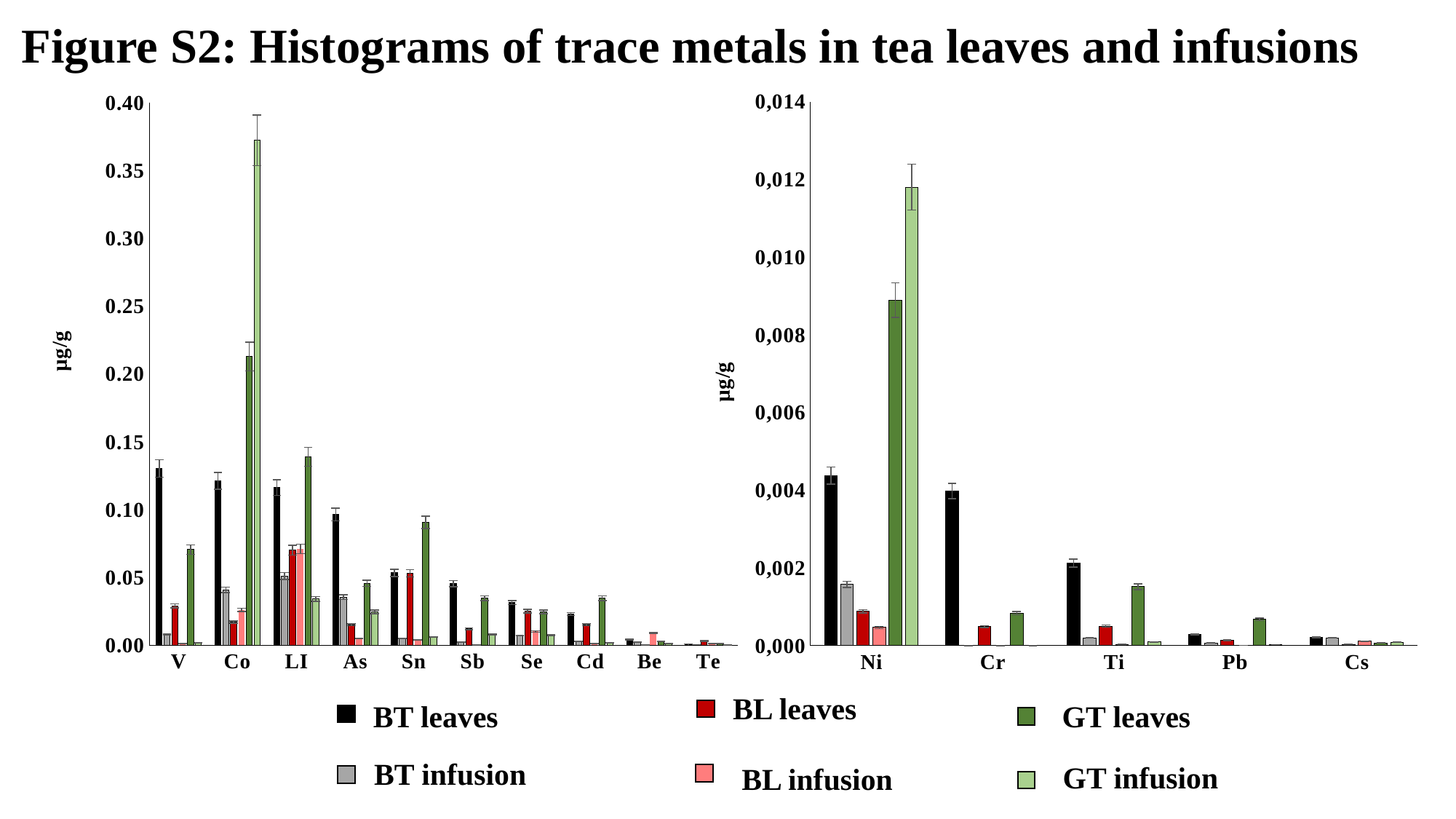

Figure S2: Histograms of trace metals in tea leaves and infusions
### Chart
| Category | BT Leaves | BT infusion | OT Leaves | OT tea | GT Leaves | GT infusion |
|---|---|---|---|---|---|---|
| Ni | 4.380999999999999 | 1.5773333333333335 | 0.881 | 0.467 | 8.8935 | 11.8 |
| Cr | 3.978 | 0.0 | 0.487 | 0.0 | 0.833 | 0.0 |
| Ti | 2.1245000000000003 | 0.20116666666666663 | 0.503 | 0.033 | 1.518 | 0.097 |
| Pb | 0.28116666666666673 | 0.0694 | 0.138 | 0.009 | 0.6785 | 0.027 |
| Cs | 0.2193333333333333 | 0.2025 | 0.035 | 0.112 | 0.07050000000000001 | 0.088 |
### Chart
| Category | BT Leaves | BT infusion | OT Leaves | OT tea | GT Leaves | GT infusion |
|---|---|---|---|---|---|---|
| V | 0.13033333333333333 | 0.007833333333333333 | 0.029 | 0.001 | 0.0705 | 0.0015 |
| Co | 0.12116666666666666 | 0.04083333333333334 | 0.017 | 0.026 | 0.213 | 0.3725 |
| LI | 0.11616666666666668 | 0.050833333333333335 | 0.07 | 0.071 | 0.139 | 0.034 |
| As | 0.09633333333333334 | 0.035333333333333335 | 0.015 | 0.005 | 0.0455 | 0.0245 |
| Sn | 0.05333333333333332 | 0.005 | 0.053 | 0.004 | 0.0905 | 0.006 |
| Sb | 0.04533333333333334 | 0.002166666666666667 | 0.012 | 0.0 | 0.0345 | 0.008 |
| Se | 0.03133333333333333 | 0.006999999999999999 | 0.025 | 0.01 | 0.0245 | 0.0075 |
| Cd | 0.022833333333333334 | 0.0028333333333333335 | 0.015 | 0.001 | 0.0345 | 0.0015 |
| Be | 0.004166666666666667 | 0.0023333333333333335 | 0.0 | 0.009 | 0.0025 | 0.001 |
| Te | 0.0008333333333333334 | 0.0003333333333333333 | 0.003 | 0.001 | 0.001 | 0.0 |BL leaves
BT leaves
GT leaves
BT infusion
GT infusion
BL infusion
